# Amendment to Atractylenolide I enhances responsiveness to immune checkpoint blockade therapy by activating tumor antigen presentation

**DOI:** 10.1172/JCI208647

**Published:** 2026-05-01

**Authors:** Hanchen Xu, Kevin Van der Jeught, Zhuolong Zhou, Lu Zhang, Tao Yu, Yifan Sun, Yujing Li, Changlin Wan, Ka Man So, Degang Liu, Michael Frieden, Yuanzhang Fang, Amber L. Mosley, Xiaoming He, Xinna Zhang, George E. Sandusky, Yunlong Liu, Samy O. Meroueh, Chi Zhang, Aruna B. Wijeratne, Cheng Huang, Guang Ji, Xiongbin Lu

Original citation: *J Clin Invest*. 2021;131(10):e146832. https://doi.org/10.1172/JCI146832

Citation for this amendment: *J Clin Invest*. 2026;136(9):e208647. https://doi.org/10.1172/JCI208647

The *JCI* previously issued a retraction for this article following joint notification by Indiana University, The Ohio State University, and the University of Maryland, College Park of data falsification in Figures 2B and 6D and misconduct findings against Xiongbin Lu and Hanchen Xu.

An independent investigation by Longhua Hospital, Shanghai University of Traditional Chinese Medicine has now been completed. Longhua Hospital’s investigative panel determined that there were 2 errors in the use of images due to operational negligence in the version of the paper submitted; however, the panel found no evidence of data fabrication, falsification, or other forms of academic misconduct.

This article remains retracted.

## Version 1 05/01/2026

Electronic publication

